# The impact of telemedicine on pediatric type 1 diabetes management: benefits, challenges, and future directions

**DOI:** 10.3389/fendo.2024.1513166

**Published:** 2024-12-20

**Authors:** Susanna Esposito, Vanessa Sambati, Federica Fogliazza, Maria Elisabeth Street, Nicola Principi

**Affiliations:** ^1^ Pediatric Clinic, University Hospital, Department of Medicine and Surgery, University of Parma, Parma, Italy; ^2^ Università degli Studi di Milano, Milano, Italy

**Keywords:** eHealth, pediatric diabetes, digital health technologies, remote healthcare, telemedicine, type 1 diabetes

## Abstract

Telemedicine (TM) has emerged as a valuable tool in managing pediatric type 1 diabetes (T1D), particularly during the COVID-19 pandemic when traditional in-person visits were limited. This narrative review examines the impact of TM on patient-provider relationships, glycemic control, and overall diabetes management in children and adolescents with T1D. Studies consistently demonstrate high levels of patient and provider satisfaction with TM, citing increased consultation frequency, reduced travel burdens, and lower associated costs. However, results regarding the effect of TM on glycemic control, as measured by HbA1c levels, are inconsistent. Some studies show significant reductions in HbA1c levels with TM use, while others report outcomes comparable to or less effective than traditional care. The effectiveness of TM also appears to be influenced by the concurrent use of advanced diabetes technologies, such as continuous glucose monitors and automated insulin delivery systems. Furthermore, TM’s impact on quality of life and other clinical outcomes beyond glucose management remains underexplored. Methodological limitations, including inconsistent randomization strategies and lack of long-term follow-up, hinder definitive conclusions. Despite these uncertainties, TM offers several advantages, such as improved accessibility and patient engagement, which may justify its broader implementation. Future research should focus on optimizing TM approaches to enhance glycemic control and quality of life, identifying the most effective strategies for specific patient groups, and addressing technological and economic barriers. This review highlights the need for comprehensive, long-term studies to fully understand TM’s potential in pediatric T1D management and its integration into standard care practices.

## 1 Introduction

Type 1 diabetes (T1D) is a chronic autoimmune metabolic disorder characterized by a complete deficiency of insulin, leading to hyperglycemia and ketonemia (1). It is one of the most prevalent chronic illnesses affecting children and adolescents worldwide. Currently, over 1.2 million individuals under the age of 19 are diagnosed with T1D globally, and studies indicate that its incidence in pediatric populations is on the rise (2). Poor management of pediatric T1D is strongly associated with acute life-threatening events and long-term complications with significant morbidity and mortality. To minimize these risks, a comprehensive approach involving medical nutrition therapy, intensive insulin regimens, and frequent blood glucose monitoring is crucial. It is also essential that both the patient and their family receive thorough education about the disease and guidance on how to continuously assess and manage its progression. Individualized care plans are necessary, as each patient’s risk for hypoglycemia or hyperglycemia varies (3). Moreover, to ensure effective disease management direct medical supervision with regular short-term visits are recommended. Unfortunately, in pediatrics, despite recently advancements in glucose monitoring and insulin administration (4), adherence to treatment regimens is generally poor, significantly increasing incidence and severity of clinical problems.

(5) Several factors explain this finding. Among them, insufficient parent- and patient-provider communication, as satisfaction with these relationships have been found essential for good health outcomes and adherence to treatment recommendations. Unfortunately, it does not occur in many T1D cases, particularly for children living far from healthcare facilities or facing provider shortages. This situation often leads to poor access to care, resulting in inadequate diabetes management and a heightened risk of complications due to long travel distances and associated costs (6).

Telemedicine (TM), the use of electronic technology to facilitate the exchange of medical information between patients and healthcare providers (7), has been effective in addressing many issues related to the frequent need for in-person visits for chronic disease management (8). Although most research in this area has focused on adults (9–11), evidence suggests that TM may also benefit pediatric patients with chronic conditions (12–15), including T1D (16–20). However, despite promising results indicating TM’s potential as an alternative healthcare delivery model, challenges remain, particularly concerning its effectiveness in managing glycemic control in children and adolescents with T1D. Not all issues associated with TM use in this demographic have been fully resolved.

This narrative review explores the current knowledge surrounding TM use in pediatric T1D management.

## 2 Methods

To reach the goal, a comprehensive review of literature was conducted using the MEDLINE/PubMed database, covering studies published from January 2000 to May 15, 2024. This time window was selected to capture the period during which TM has been developed and progressively used. The review included randomized placebo-controlled trials, controlled clinical trials, double-blind randomized controlled studies, systematic reviews, and meta-analyses. Exclusion criteria included non-English language papers, studies with insufficient data, non-peer-reviewed articles, duplicated, unavailable full texts, or abstract-only papers. The search strategy used combinations of keywords such as “Diabetes Mellitus, Type 1” AND (“Telemedicine” OR “Telemetry” OR “Telenursing” OR “Internet-Based Intervention”) AND (“Child” OR “Adolescent”), as well as “type 1 diabetes” AND “Telemedicine” OR “Telehealth” OR “children” OR “adolescents” OR “glucose metabolism.” All studies identified were assessed for relevance by VS and NP to the review based on the title and abstract. For studies that appeared to meet the inclusion criteria, or in cases when a definite decision could not be made based on the title and/or abstract alone, the full paper was obtained for detailed assessment by two researchers against the inclusion criteria. Any disagreement was resolved by consultation with a third independent reviewer (SE). The collected data were grouped and discussed according to their importance in defining the use of the different types of TM by families, patients and health providers, the relationship created between these users, and, finally, the impact of TM on the management of pediatric T1D.

## 3 Telemedicine characteristics and attitudes towards its use

### 3.1 Types of telemedicine

TM employs various telecommunication methods to facilitate the exchange of medical information between patients and healthcare providers (**Table 1**). These methods are designed to enhance the physician-patient relationship, support care plans, and promote prevention of complications and exacerbations by adhering to clinical guidelines and strategies aimed at empowering patients (20, 21). Additionally, TM provides a more comprehensive evaluation of the clinical, social, and economic needs of patients, potentially offering better health outcomes compared to traditional periodic in-person visits (22). TM methods are categorized as either asynchronous or synchronous, depending on whether they provide real-time, face-to-face interactions (23). Asynchronous communications include email, internet, cell phone, and automated messaging systems. In contrast, synchronous communications involve real-time, face-to-face interactions via videoconferencing tools such as television, digital cameras, webcams, and videophones. These technologies enable simultaneous connections between caregivers and multiple patients, offering a significant advantage, especially for patient and parent education.

**Table 1:** Telemedicine modalities and their applications in pediatric type 1 diabetes (T1D) care.

TM Modality	Description	Applications in T1D Care	Advantages	Limitations
Asynchronous (Store-and-Forward)	Involves non-real-time communication such as email, text messaging, or app-based updates.	Sharing blood glucose levels, medication logs, and progress reports with healthcare providers.	Convenient, does not require simultaneous availability of patient and provider.	Delayed response time; lacks real-time interaction.
Synchronous (Real-Time Video Consultations)	Real-time, face-to-face interactions via platforms like Zoom, Skype, or specialized healthcare software.	Conducting medical check-ups, adjusting insulin doses, and providing education and support.	Closely replicates in-person visits; allows for visual assessment and direct communication.	Requires reliable internet access and familiarity with technology; scheduling challenges.
Remote Monitoring Systems	Continuous data transmission using devices like CGM (Continuous Glucose Monitors) and automated insulin pumps.	Monitoring glucose levels and insulin delivery remotely, allowing for real-time adjustments by healthcare providers.	Provides real-time data; supports proactive management and rapid response to glucose fluctuations.	Technology can be expensive; risk of data privacy issues; requires integration with TM platforms.
Mobile Health (mHealth) Apps	Smartphone applications designed for tracking blood glucose, medication adherence, and physical activity.	Tracking patient adherence to diabetes management plans and sending automated reminders.	User-friendly and widely accessible; enhances patient engagement and self-management.	Limited functionality compared to other TM methods; not suitable for complex medical evaluations.
Telephone Consultations	Traditional phone calls between patients and providers for updates and advice.	Brief consultations, discussing test results, or providing quick medical advice.	Easy to implement; does not require advanced technology.	Lacks visual component; less effective for complex assessments and education.

TM, Telemedicine; CGM, Continuous Glucose Monitor.

### 3.2 Adoption of telemedicne

Before the COVID-19 pandemic, the use of TM in pediatric T1D care was limited, with telephone being the most common method of communication. A Canadian study found that, prior to the pandemic, TM accounted for less than 5% of all consultations and was primarily used for patients who faced difficulties accessing specialists in person (24). However, due to restrictions implemented by health authorities to limit the spread of SARS-CoV-2 (25), TM usage surged in the early months of the pandemic. The adoption of different technologies varied depending on their complexity. Initially, both patients and doctors faced challenges accessing the necessary equipment and familiarizing themselves with more complex TM methods. Additionally, some clinics hesitated to adopt synchronous communication methods immediately. Nevertheless, as access to technology improved and healthcare workers’ attitudes toward TM became more favorable, synchronous methods gained widespread acceptance and became a common approach in pediatric T1D care. A study assessing changing preferences for pediatric T1D care showed a significant shift towards using video visits over telephone consultations between the early and later phases of the pandemic, mainly due to their ease of use, advantages for learning, and higher interface quality (26). Face-to-face interactions increased from 46% to 92% of all TM visits. Furthermore, the increase in more advanced synchronous communication correlated with higher participation rates among children or adolescents (81% to 92%) and a greater involvement of healthcare workers in the TM visit (36% to 80%).

### 3.3 Adoption of telemedicine by families, patients and healthcare workers

Regardless of the method used, most studies report that patients and parents generally accept TM for pediatric T1D management and view it as an improvement. Bassi et al. evaluated patient and parent satisfaction after the introduction of televisits during the pandemic (27). The study found that the majority of patients and parents (74.1%) did not find the absence of in-person visits problematic and were satisfied with the TM service. The majority of respondents felt able to effectively communicate their medical concerns (89.9%) and receive high-quality care (92.4%), although satisfaction was higher among insulin pump users compared to those on multiple daily injections (92.4% vs. 82.5%; P = 0.023). Additionally, satisfaction levels were notably higher among families living farther from their healthcare providers (97.7% vs. 89.1%; P = 0.017). Similar findings were observed in other studies (24, 26, 28). Crossen et al. assessed T1D patient satisfaction with video-based care after six months of TM use and found that 94% of participants were very satisfied, citing improved access to care teams, enhanced monitoring of blood glucose levels, and increased knowledge of diabetes technology (29). Other studies highlighted TM’s benefits, including more comprehensive metabolic data transmission and better communication between caregivers and peers (30–34). Moreover, TM was shown to reduce diabetes-related stress, depressive symptoms, and hypoglycemia anxiety in parents of children with T1D. In a video-based telehealth intervention called Cognitive Adaptations to Reduce Emotional Stress (CARES), 41 parents of children with T1D participated in an 8–12-week program. The results indicated significant reductions in depressive symptoms and hypoglycemia fears, both at the end of the intervention and three months later.

From the perspective of diabetes specialists, including diabetologists and diabetes nurses, studies indicate that TM is considered a valuable tool for improving patient care. It allows for more frequent contacts, timely monitoring, and precise adjustments in therapy (35). However, achieving these benefits depends on healthcare providers’ familiarity with technology, a positive attitude towards virtual consultations, and a consensus on the roles and responsibilities of all professionals involved in patient care.

The minimal number of patients and endocrinologists who opted to discontinue TM after its introduction highlights the generally positive attitudes toward this intervention. In a few cases, challenges to TM adoption included concerns over privacy due to the use of shared platforms rather than personal devices, lack of integration between TM systems and existing electronic health records, and the use of technological devices that were not engaging for children and adolescents. Furthermore, the financial burden of acquiring necessary equipment and covering provider costs posed significant barriers for economically disadvantaged populations, particularly in regions where healthcare systems do not provide free communication services (36–38).

### 3.4 Unsolved problems regarding satisfaction with telemedicine

Despite these favorable findings, some questions remain unanswered regarding patient, family, and healthcare provider satisfaction with TM for pediatric T1D management. One issue is determining the best way to organize TM services. A recent systematic review and meta-analysis of 20 studies published before May 1, 2023, involving 1,704 children and adolescents with T1D (mean age 13.5 years, median diabetes duration at baseline 6.2 years) found significant heterogeneity among the studies (38). Various forms of TM were used, with differing types of information exchanged and diverse participants involved in the communication process. In 30% of the studies, smart wearable devices were employed, while 25% used smartphone applications, 15% utilized modems, 15% engaged in web-based videoconferencing, and 10% relied on telephone; 5% did not report this information. Healthcare providers used smartphone applications in 25%, websites in 15%, web conferences in 15%, telephone in 15%, and SMS text messaging in 15%. This variety created numerous combinations. Additionally, satisfaction assessments varied, with patients’ opinions often mediated by parents (in about half of the cases) and healthcare professionals’ feedback provided by individual providers in 50% of the studies, while the remaining studies involved a specialist diabetes care team consisting of diabetologists, nurses, dietitians, and psychologists. TM interventions addressed various aspects, including glucose monitoring, insulin dose adjustments, health information dissemination, and physical activity guidance, either individually or in combination (39). These limitations prevent firm conclusions regarding the best and most effective way to organize TM services for pediatric T1D care. Further limitations arise from the short duration of outcome monitoring in most studies, making it unclear whether TM satisfaction persists beyond periods when in-person visits are restricted. It is important to note that most studies on TM were conducted during the COVID-19 pandemic, and information on the impact of TM on younger children is limited. Consequently, there is insufficient evidence to determine whether TM effectively addresses the unique needs of children with T1D.

## 4 Impact of telemedicine on the patient-healthcare provider relationship

Studies examining the impact of TM on interactions between pediatric T1D patients and healthcare providers generally show high levels of satisfaction with remote consultations. The findings reveal that TM introduces several advantages, significantly enhancing patient-caregiver interactions. One notable benefit is the increase in the frequency of consultations, often reaching or surpassing the levels recommended by professional medical organizations. For example, a study involving 54 pediatric patients (mean age 12.1 ± 4.1 years) tracked over 12 months before and after the introduction of TM demonstrated an increase in specialist contacts from 2.0 ± 1.3 to 2.9 ± 1.3 per year (P = 0.0001), which aligns closely with the recommendations of the American Diabetes Association (ADA) (40). Additionally, patients and their families experienced fewer disruptions to school and work, and financial burdens associated with attending appointments were reduced.

Similar outcomes were reported in other studies, including one by Kaushal et al., who evaluated 555 patients with a mean age of 12.3 ± 3.4 years. Their research showed that TM led to an increase in the frequency of diabetes visits from 3.8 ± 1.7 to 4.3 ± 2.2 per year (P < 0.001), with 92% of these visits conducted virtually (41).

From the outset, TM’s ability to reduce time and financial burdens for patients and families was evident, even when asynchronous technologies were initially used. For example, Chase et al. assessed the impact of biweekly blood glucose data transmission using the Acculink modem over six months in a group of adolescents, without any associated in-person visits during this period (42). Compared to controls, who attended three clinic visits (at 0, 3, and 6 months) and had the option to telephone or fax blood glucose results as needed, the TM group experienced significantly lower overall expenses (P < 0.001) and fewer school absences, averaging 0.4 days per visit. Additionally, parents in the TM group did not miss working days, whereas parents of children receiving standard care missed an average of 0.5 days per clinical visit. Specifically, the mean cost per patient for the modem-based approach was $163, covering modem and cable costs, provider training, patient education, and computer use. In contrast, the mean cost per patient for traditional care was $305, including clinic visits, travel, and accommodation when necessary.

More recent data confirm the time-saving and economic benefits of TM. Crossen et al. calculated that the median duration of a video visit, including time spent uploading diabetes device data, was 33 minutes (range: 12–110 minutes). In comparison, in-person clinic visits had a median duration of 240 minutes, ranging from 120 to 4,320 minutes (29). This equated to an average time savings of 186 minutes per visit (range: 70–4,274 minutes), highlighting TM’s efficiency and its potential to significantly reduce the time burden for patients and families.

## 5 Impact of telemedicine on type 1 diabetes management

Studies investigating the impact of TM on the management of T1D suggest that the increased frequency of patient-provider interactions through TM may contribute to improved glycemic control and reductions in HbA1c levels (**Table 2**). HbA1c concentration is the gold standard for assessing long-term blood glucose control, and elevated HbA1c levels above 58 mmol/mol (7.5%) are consistently associated with negative outcomes in T1D (43). Accordingly, most studies on the impact of TM on T1D management have monitored changes in HbA1c levels during remote consultations. However, the results have been inconsistent. Some studies have shown that TM use leads to significant reductions in HbA1c levels, closely correlated with the frequency of remote consultations (44–48). In contrast, other studies have found TM to be as effective as, or sometimes less effective than, traditional in-person visits for managing glucose levels (16, 49–54). This variability makes it difficult to draw definitive conclusions about the efficacy of TM in improving glycemic control.

**Table 2:** Summary of main studies investigating the impact of telemedicine on glycemic control in pediatric type 1 diabetes.

Study	Sample Size	Age Range (Years)	TM Modality	Duration	HbA1c Reduction	Other Outcomes
Franklin et al.	200	8 – 17	Video consultations	12 months	Significant decrease (p <0.05)	Improved patient engagement
Kaushal et al.	555	12.3 ± 3.4	Virtual visits	6 months	Moderate reduction (p <0.001)	Reduced school and work absenteeism
Zhang et al.	1,704	6 – 18	Mixed modalities	3-50 months	Varies (0.22 decrease overall)	Higher satisfaction in pump users
Crossen et al.	150	10 - 16	Video-based care	6 months	No significant change	Significant time savings
Chase et al.	80	13 - 18	Bi-weekly data upload	6 months	Slight decrease (NS)	Reduced overall expenses (p< 0.001)

NS, Not significant; TM, Telemedicine.

Conflicting results also arise when evaluating other glucose management parameters besides HbA1c. For example, a meta-analysis showed that while TM was associated with a reduction in hypoglycemia incidence compared to controls [mean difference (MD) –0.15, 95% CI –0.57 to 0.27; P = 0.49], the difference was not statistically significant, making it unclear whether TM had a meaningful impact (51). Further doubts about the efficacy of TM emerge when considering the methodological limitations of studies on HbA1c variations. Although many of these studies were randomized clinical trials, only 25% adequately described their randomization strategies and allocation concealment. Additionally, many studies failed to report on withdrawals and dropouts, weakening the reliability of their findings.

Moreover, other factors, aside from TM, may have contributed to observed reductions in HbA1c levels. For instance, Franklin et al. suggested that the HbA1c improvements in their study were likely due to the intensity of insulin treatment rather than TM alone (53). Similarly, Zhang et al.’s systematic review and meta-analysis reported that HbA1c levels were assessed at intervals ranging from 3 to 50 months post-TM introduction (38). A global evaluation indicated a significant reduction in HbA1c levels by 0.22 (95% CI –0.33 to –0.10; P < 0.001) with TM. However, subgroup analysis revealed that TM was not effective at 3 months (MD –0.30, 95% CI –0.62 to 0.02; P = 0.07) or at 12 months (MD 0.04, 95% CI –0.33 to 0.40; P = 0.85). A significant improvement was only observed at the 6-month mark (MD –0.21, 95% CI –0.37 to –0.05; P = 0.01). This suggests TM’s effectiveness may peak around 6 months, but it may diminish over time. Alternatively, these HbA1c trends could be influenced by the so-called honeymoon phase of T1D, where patients experience a temporary period of remission with lower insulin needs and stable HbA1c levels, typically beginning about 3 months after the onset of insulin therapy and lasting several months (54).

The improvements in glycemic control observed with TM could also be attributed to concurrent use of advanced technologies such as continuous glucose monitors (CGM) and insulin pumps. Studies have shown that these devices, especially when integrated into automated insulin delivery systems, can significantly enhance glycemic control in both adults and children. For example, a recent multicenter, open-label randomized controlled trial involving 80 pediatric patients with poorly controlled T1D (HbA1c ≥8.5%) demonstrated that an automated insulin delivery system significantly reduced HbA1c levels from 10.5 ± 1.9% to 8.1 ± 1.8% after 13 weeks, whereas the control group saw no improvement (HbA1c remained at 10.4 ± 1.6% to 10.6 ± 1.8%). Moreover, no significant adverse events occurred in the TM group, while the control group reported one case of severe hypoglycemia and two cases of diabetic ketoacidosis (51). The role of these new technologies is further supported by evidence showing that studies where TM was less effective were conducted before 2010, a period when such technologies were not widely utilized. In contrast, studies with positive results were carried out more recently, coinciding with the increased adoption of CGM and insulin pumps (55).

Furthermore, TM studies provide limited information on its impact beyond glycemic control in pediatric T1D management. While insulin dosage adjustment, dietary advice, physical activity guidance, and health education are common aspects of TM, clinical outcomes beyond blood glucose levels are seldom reported. For instance, information on blood pressure, weight, and other relevant clinical markers is often lacking. Only a few studies have assessed the impact of TM on quality of life (QoL), and even these provide inconsistent results. The use of the Diabetes Quality of Life for Youth score did not show a significant difference between TM and usual care (impact of diabetes: P = 0.59; diabetes-related worries: P = 0.71; satisfaction with diabetes: P = 0.68) (56). Conversely, studies using non–youth-specific QoL indicators, such as the frequency of self-monitoring blood glucose and hypoglycemia incidence, reported statistically significant improvements (MD –0.24, 95% CI –0.45 to –0.02; P = 0.04) (57).

## 6 Conclusions

T1D management is one of the areas where TM has the potential to provide substantial benefits for patients, families, and healthcare systems. **Table 3** summarizes benefits and challenges of TM in pediatric T1D. Overall, despite limitations previously reported, available studies suggest that TM can improve T1D care when both patients and providers are familiar with the technology and maintain a positive attitude towards remote consultations (38, 56). Under these conditions, TM increases patient-provider interactions, matching or even exceeding the frequency of traditional in-person visits. This results in various benefits, such as reduced travel-related burdens, time savings, and decreased financial costs for families. More frequent communication also helps patients and families better understand the disease, its monitoring requirements, and how to effectively use glucose monitoring and insulin infusion devices. Additionally, healthcare providers can offer more timely and precise medical monitoring and therapy adjustments through TM.

**Table 3:** Benefits and challenges of telemedicine in pediatric T1D management.

Benefits		Description
Increased Frequency of Consultations		TM increases the frequency of patient-provider interactions, often meeting or exceeding ADA guidelines.
Reduced Travel Burdens		Virtual consultations reduce the need for travel, saving time and costs for families.
Enhanced Patient Engagement		TM facilitates more frequent monitoring, empowering patients and families in diabetes management.
Integration with Advanced Diabetes Technology		TM works effectively with devices like CGM and insulin pumps to optimize glucose control.
High Patient and Provider Satisfaction		Studies report overall satisfaction with TM, especially among families living far from healthcare centers.

Challenges		Description
Inconsistent Impact on Glycemic Control	Mixed evidence on whether TM effectively reduces HbA1c levels compared to traditional in-person visits.	
Technology Access and Familiarization	Both patients and providers may face difficulties accessing and learning to use necessary technologies.	
Economic Barriers	Out-of-pocket expenses for equipment and provider fees may limit access, especially in underserved areas.	
Limited Long-Term Data	Few studies provide long-term data on TM’s impact, raising questions about its sustainability.	
Privacy and Data Security Concerns	Using shared platforms instead of personal devices may raise privacy issues among patients and families.	

Despite these benefits, studies remain too generic to identify the most effective methods for maximizing satisfaction among patients and healthcare providers. Non-glucose-related clinical parameters such as blood pressure and weight, which are vital for holistic T1D management, often remain unaddressed during TM visits. Additionally, essential components of diabetes care—such as pubertal staging, examination of injection sites for lipohypertrophy or infection, and physical screening for complications (e.g., foot exams or retinopathy evaluations)—are either suboptimal or entirely absent in the TM setting. This lack of hands-on assessment may lead to delayed identification and intervention for potential complications. Therefore, a balanced approach combining in-person visits and telemedicine is critical to ensure comprehensive care. In-person visits can facilitate thorough physical examinations, structured screenings, and interpersonal connection, while TM offers convenience and increased frequency of interaction for addressing glucose management, education, and psychosocial support. By integrating these modalities, healthcare providers can leverage the strengths of both approaches, ensuring optimal outcomes while addressing the logistical challenges often faced by patients and their families. Moreover, studiesdo not sufficiently address the challenges of different TM approaches. Further research is needed to identify the optimal TM approach tailored to each patient’s needs, family circumstances, and available medical support. Establishing an ideal TM model is essential not only to enhance the already observed benefits but also to determine under which conditions TM can positively influence the T1D disease course. Current data do not definitively establish whether TM provides glycemic control comparable to that achieved through traditional in-person care. If equivalent control is confirmed, the social and economic advantages TM offers would make it a compelling option for the management of pediatric T1D, justifying its wider adoption in treating children and adolescents with this condition. Furthermore, future studies should assess the significance of potential challenges associated with telemedicine in managing T1D, including concerns about the privacy and security of collected data, limited access to reliable internet and technology, and the economic burden of implementing and maintaining these systems.
